# Pulmonary Vein Stenosis: Moving From Past Pessimism to Future Optimism

**DOI:** 10.3389/fped.2021.747812

**Published:** 2021-10-05

**Authors:** Daniel I. McLennan, Elyan C. Ruiz Solano, Stephanie S. Handler, Joy Lincoln, Michael E. Mitchell, Edward C. Kirkpatrick

**Affiliations:** ^1^Section of Pediatric Cardiology, Department of Pediatrics, Medical College of Wisconsin, Milwaukee, WI, United States; ^2^Herma Heart Institute, Children's Wisconsin, Milwaukee, WI, United States; ^3^Section of Cardiothoracic Surgery, Department of Surgery, Medical College of Wisconsin, Milwaukee, WI, United States

**Keywords:** pulmonary vein stenosis, cardiac catheterization, congenital heart disease, congenital cardiothoracic surgery, sutureless pulmonary vein repair, pulmonary vein stent angioplasty

## Abstract

Pulmonary Vein Stenosis (PVS) is a rare disease with a prevalence of around 1. 7 cases per 100,000 children under 2 years old. Treatment options for this disease have not provided great results and pathophysiology of this condition is still poorly understood. Here, we will review the history of PVS including diagnostic tools and treatments, the current management approach, and what the future holds for this devastating disease.

## Introduction

As a junior cardiology trainee, one was often taught that “pulmonary vein stenosis (PVS) is a terrible disease, affected individuals all die and there is little point to try and intervene on them.” With mortality rates in the literature as high as 60% at 2 years after diagnosis (1), and little historic information about the pathogenesis and best treatment, this has continued to be the general philosophy for this condition. However, as the field better understands the pathobiology of this disease, and therefore improved treatment options become more available, the overall outlook for this condition has changed. Here we will explore the history, the present, and the future of PVS.

## History of Pulmonary Vein Stenosis

PVS is a rare disease with a prevalence of around 1.7 cases per 100,000 children under 2 years old (2). PVS was first described in the literature by Dr. Reye from Sydney Australia in June 1951 (3), and was recognized early as a severely progressive and fatal disorder. Not much was known about the pathology, cause, or treatment for many years, until in 1971 when Dr. Kawashima from Osaka Japan performed the first direct PVS surgical repair on a patient (4).

In parallel with surgical approaches, cardiac catheterization inventions were being pursued to diagnose and treat PVS. By the 1970s angiography was commonplace in the diagnosis of PVS (20). In April of 1980, a team of interventional cardiologists from Texas children's hospital performed the first documented pulmonary vein angioplasty in a 35-year-old woman with sclerosing mediastinitis through a hybrid sternotomy approach. She died 9 months later, but this demonstrated an important beginning for PVS catheter-based interventions (5).

Throughout the 1980's balloon intervention on the pulmonary veins continued with little change in outcome. However, in the late 1980s, the teams at Texas Children's and Boston Children's hospitals began to evaluate the potential for PVS stenting. The first case described an unsuccessful stent embolization, however, the second described an improved catheter-based stent implantation in the right and left pulmonary veins after surgical repair of TAPVR (6). Around the same time, a clinical team at the University of Michigan had experimented with surgical and percutaneous implantation of a stainless steel Palmaz stent (Cordis Europa N.V, the Netherlands) into the pulmonary veins of three children. While pressure gradients dropped from 11 mmHg to less than one in all stented pulmonary veins, the effects were short-lived as re-stenosis occurred by 6 months (7). However, the benefits of short-term relief experienced by stent implantation initiated a positive change in clinical management that is used today.

In parallel with cardiac catheterization approaches, surgical teams continued to explore additional PVS treatment strategies. In 1985 Pacifico et al. described a technique where living autologous tissue from the left atrial appendage was used as an onlay patch to widen the stenotic area and create a double pathway for venous return (21). Then in 1996 in France and 1998, in Toronto Canada, the atriopericardial repair or “sutureless technique” for PVS was first performed. They described using the patient's pericardium to form a left atrium extension and reduce the trauma directly to the pulmonary vein (8). They also applied this technique for Total anomalous pulmonary vein return (TAPVR) repair (9). This appeared to provide better long-term outcomes for these patients, but with so little known about the disease, these outcomes still had room for improvement.

### Current Day Approach to Pulmonary Vein Stenosis

In the last two decades, the appreciation for the pathophysiology and treatment of PVS has grown. Through advances in the understanding of the disease process, PVS is now considered present in multiple forms that are largely classified as primary or secondary. Primary PVS is a condition where patients present with disease phenotypes without a history of pulmonary vein intervention or injury. This is felt to be the result of abnormal incorporation of the pulmonary veins into the left atrium. Conditions associated with primary PVS include prematurity, bronchopulmonary dysplasia (BPD), genetic syndromes, low birth weight, and congenital heart disease such as patent ductus arteriosus, atrial and ventricular septal defects (12, 13). The condition appears to evolve in patients with prematurity and BPD during infancy with most patients having at least one normal echocardiogram before the condition is detected by a median age of around 7 months (15). Two-year survival in one cohort of premature infants was 43% with higher mortality associated with younger age at diagnosis (<6 months), being small for gestational age, and increase number of veins involved (>2 veins) (15). Stenosis of the pulmonary veins may appear as a relatively discrete shelf at the atrial junction, upstream extension into the pulmonary vein, or as diffuse hypoplasia of the pulmonary vein (14, 17).

In contrast to primary PVS, secondary PVS occurs after intervention or injury to the pulmonary veins. The most common occurrence is after the repair of total anomalous pulmonary venous return (TAPVR) that can occur in 10% of patients (9) making up to 76% of secondary PVS (18). In the adult population, there has been an increase in secondary PVS as a complication of radiofrequency ablation in the left atrium, usually performed for atrial fibrillation (17). Other forms seen in adults are secondary to malignancies or their treatments and other inflammatory processes like sclerosing mediastinitis (18).

With the knowledge that TAPVR had higher numbers of secondary PVS, the sutureless technique for surgical repair was adopted as previously mentioned for both the primary repair of TAPVR as well as secondary PVS. This technique has also been adopted for the repair of primary PVS. In a recent review of the sutureless repair vs. primary venous anastomosis, Sutureless repair had less restenosis (40 vs. 67%), less reintervention (31 vs. 61%) less mortality (20 vs. 33%) these results lean toward longer freedom from re-intervention and survival with the sutureless technique (22). For this reason, most centers have moved toward this approach as their primary option for PVS surgical repair.

Despite the improved surgical successes, there was still a significant failure, with between 25 and 50% of sutureless repairs having restenosis (22, 23). The role of the cardiac catheterization laboratory, therefore, became important in trying to improve further the outcomes of these patients. As stated, the current balloon and stent techniques had improved outcomes, but with 5-year survival between 30 and 50% (24, 25) with severe PVS, more needed to be done to improve long-term survival.

The team at Emory in Atlanta took an aggressive approach to cardiac catheterization in PVS to prevent vein loss and improve outcomes. The hypothesis was that regular catheter therapy would provide better lasting results than only intervening once or when the patient was symptomatic. Outcomes showed that regular vein intervention improved the survival from 36 to 80% (24), and demonstrated that regular surveillance with echo and catheter allowed for earlier identification of re-stenosis and therefore ability for earlier intervention. Furthermore, for patients who were treated once with no re-intervention, the survival was <30% and for those who had regular reintervention, the survival at 1 year was >80% (24).

The use of bare-metal stents (BMS) in PVS had been shown to improve vein stenosis and lumen size (26). Unfortunately, there was often severe early re-stenosis. The group from Boston Children's showed that with BMS, 5-year survivability was <50% (26). With the evolution of the drug-eluting stent (DES) and balloon in adult interventional cardiology, the thought was that this new therapy could have a role in PVS. The groups in Atlanta and Texas showed that with implantation of DES the survival of patients with infant PVS increased up to 60–80% (24, 25). The Emory group showed that DES survivability was 68% and BMS 33%, more than halving the mortality for this group. Drug-eluting stents are only available up to 5 mm, with expansion ability to 6.25 mm before they reach their expansion limit6. The group from Boston had shown that once a pulmonary vein is stented >7 mm the rate of in-stent stenosis is reduced to <30% at 1 year compared to 70% below 7 mm (26).

The improvement in surgical repair of PVS and newer stent technology mixed with aggressive re-intervention has changed the trajectory for these patients tremendously, but with mortality rates of 30–40% still predicted at 5 years post intervention (15, 25, 27) more still needs to be done. There is a body of emerging evidence that a more constant immune-modulating therapy might be useful in PVS, this is thought due to evidence from both animal and human pulmonary vein biopsies, that fibroblast cells replace endothelial cells in the lumens of obstructed pulmonary veins. Is this the future for further PVS treatment to improve outcomes?

### The Future of Pulmonary Vein Stenosis Treatment

Attempts to find better therapeutic options for PVS have led to the identification of the histopathological changes that are unique to PVS. It has been demonstrated that stenotic pulmonary vein intimal hyperplasia is due to tissue proliferation of a type of spindle cells with smooth muscle-like properties classified as myofibroblasts (10, 11). This led to the theory of treating PVS as a neoproliferative condition with chemotherapy to halt myofibroblastic proliferation (10). However, while an open-label trial using vincristine and methotrexate to treat PVS in congenital heart disease patients showed disease stabilization in 33% of patients and 38% survival after 1 year, survival rates were 0% in patients with PVS alone (12). Mild to moderate side-effects from therapy were common (12). The disappointing results then led to a revised open-label protocol using the tyrosine kinase inhibitors imatinib ± bevacizumab to target myofibroblast neoproliferation (13). This medical regimen with the addition of pulmonary vein interventions both surgical and catheter-based did manage to have 77% of patients survive 72 weeks since therapy onset (13).

Using a piglet model, the group from Toronto was able to reproduce extra-pulmonary PVS by banding individual pulmonary veins and then evaluated the effects upon intrapulmonary (“upstream”) veins (14). They found that localized PVS promoted intimal fibromuscular hyperplasia and arterialization of the intrapulmonary veins (14). Concurrently there was evidence of endothelial to mesenchymal transformation through cell marker analysis that appeared to be driven by transforming growth factor-β1 (TGF- β1) expression creating a cellular conversion to myofibroblasts in intrapulmonary veins (14). This piglet model was later used to test the effects of losartan, an angiotensin II receptor blocker that also indirectly blocks TGF- β1 (15, 16) on PVS remodeling. The results did show some decrease in pulmonary vein intimal hyperplasia, but no effects on TGF- β1 levels (15). However, when a stent was placed in a piglet pulmonary vein after 3 weeks of pulmonary vein banding, TGF-β1 levels were lower than in non-stented PVS piglets implying that at least partial reversal of endothelial to mesenchymal transformation is possible with stenting alone (14). Another piglet model of PVS also demonstrated local and “upstream” pulmonary vein intimal changes from “dedifferentiated” smooth muscle cells [Masaki]. This group was able to show an increase in activation of the mTOR pathway that leads to smooth muscle-like cell (i.e., myofibroblast) migration and proliferation which was suppressed by the mTOR inhibitor rapamycin [Masaki]. Clinically the mTOR inhibitor sirolimus has been used to decrease the mTOR upregulation to slow the rate of in-stent restenosis after pulmonary vein intervention (17, 28). This drug effect appears to benefit more rapid re-stenosing veins and veins with larger placed stents (>7 mm) (17, 28) and may have survival benefit in the setting of aggressive catheter based interventions (28).

However, an explanation of the etiology of myofibroblasts proliferation in PVS is still lacking. In collaboration with colleagues at MIT, our group at Children's Wisconsin found that in PVS biopsy specimens, there was the presence of a unique multipotent stem cell known as a metakaryotic cell (18). This cell type is characterized by its bell-shaped nuclei and amitotic division (18). It is an early evolutionary cell that falls between prokaryotic and eukaryotic cells based upon its nuclear organization (18). It was shown that these cells are the likely precursors to myofibroblasts (18). In addition, they are resistant to several types of chemotherapeutic drugs which may explain the lack of success in using them for PVS in the past (18). However, metakaryotic cells appear sensitive to other drugs such as metformin which was shown to have the potential to slow the progression of PVS (19).

The clinical implications of our current understanding of the pathophysiology and treatment outcomes of PVS are just now being realized. This has led to some academic centers creating focused programs on PVS management that use a multidisciplinary approach which can incorporate translational research as well as medical, surgical, and catheter-based treatment options. In addition, the use of multi-institutional registries, such as the PVS Network, will serve as important sources of reviewing patient outcomes to advance research and clinical care of PVS.

## Conclusion

By learning from our past experiences, we are beginning to see new hope in combating this devastating disease. Newer surgical and catheter-based approaches are beginning to show some improvements in the outcome that are further enhanced with targeted drug therapy. An aggressive multidisciplinary approach may be the most successful way to address PVS. By having a diverse team made up of specialists (cardiothoracic surgeons, interventional cardiologists, pulmonary hypertension) with interest in PVS, it will allow for the most up to date approach to each individual patient with PVS. It is clear that PVS is not a disease where one approach is suited for each patient. What therapeutic pathways are used depends upon several factors including the mechanism of PVS, patient co-morbidities and institutional resources. Due to this complexity, a targeted pulmonary vein stenosis working group within an institution can be utilized to create the best treatment options for an individual patient. that will likely lead to a better future for these patients.

## Author Contributions

DM, ER, and EK were responsible for the body of the manuscript, for reviewing and editing. SH, MM, and JL were responsible for editing and reviewing the manuscript. All authors contributed to the article and approved the submitted version.

## Conflict of Interest

The authors declare that the research was conducted in the absence of any commercial or financial relationships that could be construed as a potential conflict of interest. The Reviewer DI declared a past collaboration with several of the authors DM and SH to the handling editor.

## Publisher's Note

All claims expressed in this article are solely those of the authors and do not necessarily represent those of their affiliated organizations, or those of the publisher, the editors and the reviewers. Any product that may be evaluated in this article, or claim that may be made by its manufacturer, is not guaranteed or endorsed by the publisher.
